# Investigation of the chaperone function of the small heat shock protein — AgsA

**DOI:** 10.1186/1471-2091-11-27

**Published:** 2010-07-24

**Authors:** Toshifumi Tomoyasu, Atsushi Tabata, Hideaki Nagamune

**Affiliations:** 1 Department of Biological Science and Technology, Institute of Technology and Science, The University of Tokushima Graduate School, Minami-josanjima-cho, Tokushima 770-8506, Japan

## Abstract

**Background:**

A small heat shock protein AgsA was originally isolated from *Salmonella enterica *serovar Typhimurium. We previously demonstrated that AgsA was an effective chaperone that could reduce the amount of heat-aggregated proteins in an *Escherichia coli rpoH *mutant. AgsA appeared to promote survival at lethal temperatures by cooperating with other chaperones *in vivo*. To investigate the aggregation prevention mechanisms of AgsA, we constructed N- or C-terminal truncated mutants and compared their properties with wild type AgsA.

**Results:**

AgsA showed significant overall homology to wheat sHsp16.9 allowing its three-dimensional structure to be predicted. Truncations of AgsA until the N-terminal 23^rd ^and C-terminal 11^th ^amino acid (AA) from both termini preserved its *in vivo *chaperone activity. Temperature-controlled gel filtration chromatography showed that purified AgsA could maintain large oligomeric complexes up to 50°C. Destabilization of oligomeric complexes was observed for N-terminal 11- and 17-AA truncated AgsA; C-terminal 11-AA truncated AgsA could not form large oligomeric complexes. AgsA prevented the aggregation of denatured lysozyme, malate dehydrogenase (MDH) and citrate synthase (CS) but did not prevent the aggregation of insulin at 25°C. N-terminal 17-AA truncated AgsA showed no chaperone activity towards MDH. C-terminal 11-AA truncated AgsA showed weak or no chaperone activity towards lysozyme, MDH and CS although it prevented the aggregation of insulin at 25°C. When the same amount of AgsA and C-terminal 11-AA truncated AgsA were mixed (half of respective amount required for efficient chaperone activities), good chaperone activity for all substrates and temperatures was observed. Detectable intermolecular exchanges between AgsA oligomers at 25°C were not observed using fluorescence resonance energy transfer analysis; however, significant exchanges between AgsA oligomers and C-terminal truncated AgsA were observed at 25°C.

**Conclusions:**

Our data demonstrate that AgsA has several regions necessary for efficient chaperone activity: region(s) important for lysozyme chaperone activity are located outer surface of the oligomeric complex while those region(s) important for insulin are located inside the oligomeric complex and those for MDH are located within the N-terminal arm. In addition, the equilibrium between the oligomer and the dimer structures appears to be important for its efficient chaperone activity.

## Background

The heat shock response in pathogenic bacteria is induced by a large variety of stresses including heat and the host immunodefence system [1]. Transcription of heat shock genes under stress conditions in Gram-negative bacteria is induced by the heat shock transcription factor σ^32^, encoded by *rpoH *[2,3]. Small heat shock proteins (sHsps) protect the system against the irreversible aggregation of non-native proteins and assist in their refolding by major cytosolic chaperones such as the ClpB-Hsp70 chaperone system and GroEL/GroES [4-8]. Most of the members of the *Enterobacteriaceae *family have two conserved sHsps, IbpA and IbpB [9]. Interestingly *Salmonella enterica *serovar Typhimurium has a third conserved sHsp aggregation-suppressing protein (AgsA) [10]. AgsA is a heat shock protein that is also believed to function as an effective cytosolic chaperone, because its overproduction partially complements the thermo-sensitive phenotype of the *dnaK *null mutant, and it can prevent the aggregation of denatured proteins in *dnaK *and *rpoH *null mutants. In *S. enterica *serovar Typhimurium, AgsA appears to play a role in survival at lethal temperatures by cooperating with other chaperones, including IbpA and IbpB.

Members of the sHsp family are found in most organisms. sHsps and related α-crystallins comprise a superfamily of chaperones defined by: (i) a conserved domain of 80-100 amino acids (AA), referred to as the α-crystallin domain; (ii) a short C-terminal extension, flanking this domain; (iii) an N-terminal arm of variable length and highly divergent sequence; (iv) a molecular mass typically between 12-42 kDa; (v) large oligomers of sHSPs, formed in their native state and (vi) an ATP independent chaperone activity [11-13].

Structural and biochemical studies have demonstrated that the α-crystallin domain is the basic building block of most sHsps, and it is an important domain for the interaction between sHsps and unfolded substrate proteins [12-14]. The crystal structures have been solved for *Methanococcus jannaschii *Hsp16.5 and for wheat (*Triticum aestivum*) Hsp16.9 [15,16]. Comparison of both structures has revealed that although the overall organisation of the complexes are different, both have dimer as their primary building block, and their α-crystallin domains form very similar IgG-like β-sandwich folds [16].

The N-terminal arm and C-terminal extension are believed to be important regions for regulating chaperone activity. The N-terminal arm is attached to one end of the α-crystallin domain, is variable in length and sequence among sHsps and is thought to influence higher order oligomerisation, subunit dynamics and chaperone activity [16-23]. The C-terminal extension, a charged and highly flexible region, stabilises oligomers while mediating sHsp solubility and chaperone activity (roles that are similar to those proposed for the N-terminal arm) as well as being able to form bonds with other C-terminal extensions [16,22-26].

**Figure 1:**
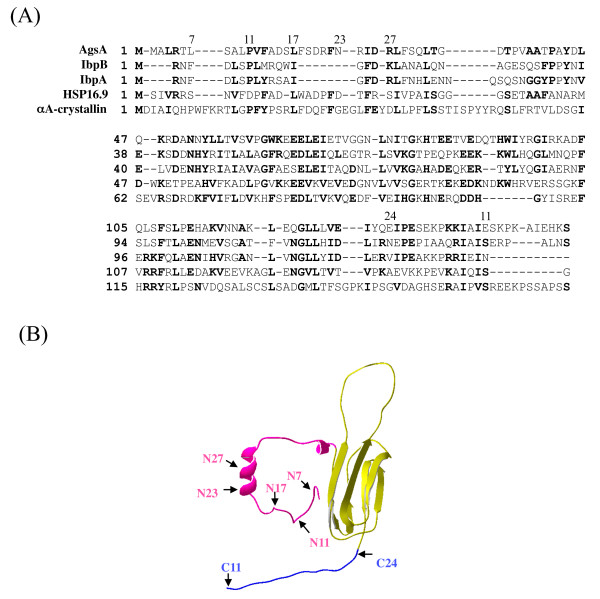
**(A) Amino-acid alignment and (B) predicted three-dimensional structure of AgsA**. (A) The alignment includes the *S. enterica *serovar Typhimurium AgsA (GenBank: GU130588), *E. coli *IbpA (GenBank: BAE77607) and IbpB (GenBank: BAE77608), *T. aestivum *Hsp16.9 (PDB: 1GMEA), and *Bos taurus *α-crystallin (GenBank: NP776714). Identical and similar (L/V/I, F/Y/W/H, R/K/H, D/E, G/A, S/T) amino acid residues are shown in bold if they occur in at least 3 sequences. (B) Structure of AgsA was predicted by the Geno3 D server (PBIL, Lyon, France) using the structure of *T. aestivum *Hsp16.9 as a template. The α-crystallin domain (yellow), N-terminal arm (red), and C-terminal extension (blue) are distinguished by colors. The arrows indicate the position of the N- or C-terminal truncations of AgsA used in this study.

The finding that AgsA shares significant and overall homology to wheat sHsp16.9 (Figure 1A) suggests that biological and biochemical studies of AgsA will inform research on both bacterial and eukaryotic sHsps. Therefore, we investigated the *in vivo *and *in vitro *chaperone activities of AgsA and N- or C-terminal truncated AgsA mutants using chemically- and heat-denatured substrates. From these studies, we elucidated that multiple regions are involved in the chaperone activity of AgsA to different denatured substrates, and that the equilibrium between the oligomeric and dimeric forms appears to be important for efficient chaperone activity.

## Results

### Multiple alignment and structural model of AgsA

Multiple alignment of AgsA to IbpA, IbpB and bovine α-crystallin showed that the α-crystallin domain of AgsA was conserved but that the N-terminal arm and the C-terminal extension were variable (Figure 1A). Interestingly, with the exception of a portion of the C-terminal extension, AgsA shared sufficient overall homology with wheat sHSP16.9 to allow prediction of its three-dimensional structure (Figure 1B). The predicted structure of AgsA showed that the N-terminal arm was composed of helices connected by a random coil, and that the α-crystallin domain consisted of IgG-like β-sandwich folds together with the short C-terminal extension. The N-terminal arm and the C-terminal extension are important regions for oligomer formation and chaperone activity in several sHsps although their contribution to these functions in AgsA has not been determined. Therefore, we constructed the following AgsA N- or C-terminal truncated mutants: (i) ΔN7, truncated up to leucine-7 which is located in front of the gap region determined by its homology to bovine α-crystallin; (ii) ΔN11, truncated up to proline-11 which is a conserved residue except in Hsp16.9; (iii) ΔN17, truncated up to leucine-17 which is conserved in all aligned sHsps; (iv) ΔN23, truncated up to asparagine-23 which is located in front of the α-helix of the N-terminal region; (v) ΔN27, truncated up to arginine-27 which contains half of the α-helix; (vi) ΔC11, truncated from glutamic acid-146 which is located just after the conserved V/IXI/V motif where sHsp form oligomeric complexes with the C-terminal extension by intermolecular interactions and (vii) ΔC24, truncated from glutamic acid-133 resulting the elimination of the whole C-terminal extension.

### *In vivo *chaperone activity of truncated AgsA

**Figure 2:**
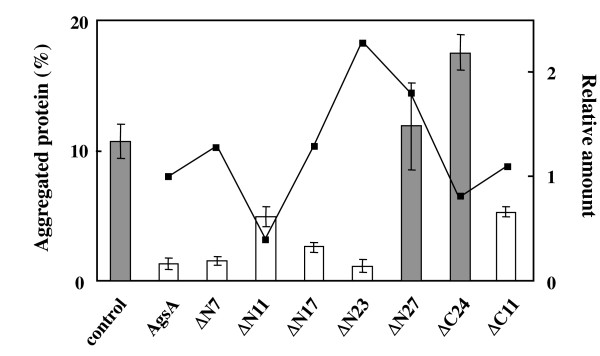
**Chaperone activity of the truncated AgsA in the Δ*rpoH *mutant**. The cells were grown in LB medium supplement with 1 mM IPTG at 30°C for 3 h and further cultured at 42°C for 1 h. Total proteins and aggregated proteins were isolated as described in the Materials and Methods. Equal amounts of total proteins (10 μg) were analyzed by 15% SDS-PAGE. The stained gels were scanned and the level of AgsA and its truncated mutants were quantified. Line graph and right ordinate show the relative amount of truncated AgsA with the amount of AgsA set to 1. Bar graph and left ordinate show the percentage of aggregated protein calculated by the ratio of the aggregated protein and the total protein. Error bars show the SD from 3 different experiments.

To investigate whether truncation of the N-terminal arm or C-terminal extension of AgsA decreases its chaperone activity, we examined the *in vivo *chaperone activity of truncated AgsA by using the Δ*rpoH *mutant [10,27]. This mutant lacks the heat shock transcription factor σ^32 ^and is therefore largely devoid of all major cytosolic chaperones, except for GroEL/GroES, and has lower levels of proteases [10,28]. The lack of chaperones and proteases results in a large accumulation of aggregated proteins, that is, approximately 10% of the total proteins were aggregated at 42°C (Figure 2). When wild type AgsA was overexpressed in Δ*rpoH *cells, the amount of aggregated proteins decreased to less than 1% of the total protein levels observed at 42°C. The overexpression levels of the truncated AgsA by IPTG induction in Δ*rpoH *cells was found to be diverse (Figure 2): ΔN11 only accumulated to approximately half the level of wild type AgsA, while other truncated proteins (ΔN7, ΔN17, ΔC24 and ΔC11) were produced to the same level as the wild type protein or higher (ΔN23 and ΔN27). Quantification of the amount of aggregated proteins in the truncated AgsA-overexpressing Δ*rpoH *cells revealed that aggregation was significantly prevented until the truncation of the 23^rd ^AA of the N-terminus, while ΔN27 did not show any chaperone activity. ΔN11 showed weaker chaperone activity, although this was caused by its inefficient expression in Δ*rpoH *cells. C-terminal deletions also affected the chaperone activity of AgsA. ΔC11 showed weaker chaperone activity than AgsA, while ΔC24 did not exhibit any chaperone activity. Thus, truncated AgsA demonstrated *in vivo *chaperone activity by the part from the 24^th ^AA of the N-terminus to the 12^nd ^AA of the C-terminus. We purified AgsA, ΔN11, ΔN17, ΔN23 and ΔC11 to investigate their oligomeric state and chaperone activity *in vitro*. However, purified ΔN23 tended to aggregate in our assay conditions preventing its use in the following experiments.

### Oligomeric status of N- or C-terminal truncated AgsA

**Figure 3:**
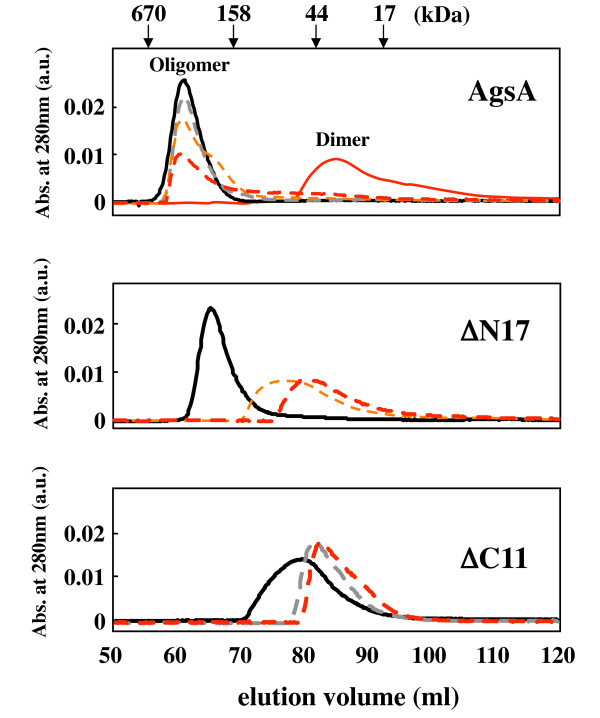
**Size exclusion chromatography of AgsA, ΔN17 and ΔC11**. The elution profiles of the proteins were recorded using size exclusion chromatography at the indicated temperature. Black solid line, 25°C; broken gray line, 37°C; broken yellow line, 42°C; broken red line, 50°C; and red solid line, 60°C. The retention volume of protein molecular weight standards and the positions of the oligomer and the dimer of AgsA are shown above the first panel.

The effects of N- or C-terminal truncations on the AgsA complex size under different temperatures were determined using size exclusion chromatography (SEC; Figure 3). The chromatograph for AgsA showed a single peak at 25°C with a calculated apparent molecular mass of 419 kDa, corresponding to a complex of approximately 22 subunits. The main peak fraction for AgsA was located at the same position (419 kDa) until 50°C. However, the amount of AgsA eluted in the lower molecular weight fractions gradually increased with increasing temperature. When SEC was carried out at 60°C, AgsA was found to largely dissociate and its molecular mass changed to approximately 29.3 kDa, corresponding to the middle size of monomer and dimer with broad shoulders. ΔN11 (data not shown) and ΔN17 eluted in the large molecular weight fractions and exhibited a single peak corresponding to approximately 21 and 15 subunits at 25°C, respectively. In contrast to AgsA, almost all ΔN11 and ΔN17 dissociated at 42°C and eluted as a single peak with broad downstream shoulders corresponding to the hexamer and tetramer, respectively. At 50°C ΔN11 and ΔN17 eluted in the fraction corresponding to the dimer. ΔC11 could not form large oligomeric complexes and eluted in the lower molecular weight fractions, corresponding to the trimer at 25°C and dimer at 37°C and 42°C.

### *In vitro *chaperone activity of AgsA and its N- or C-terminal truncated mutants

Chaperone activity of purified AgsA and its N- or C-terminal truncated mutants was assayed by monitoring the ability to prevent the aggregation of chemically denatured substrates: lysozyme and insulin (Table 1 and Additional files 1 and 2, Tables S1 and S2) or heat-denatured substrates: malate dehydrogenase (MDH) and citrate synthase (CS) (Table 2 and Additional file 3 and 4, Tables S3 and S4). We confirmed that in the absence of substrate AgsA and the N- or C-terminal truncated mutants did not increase the turbidity by aggregation at our assay temperatures (data not shown). We previously reported that the overproduction of AgsA could reduce the amount of aggregated proteins in Δ*rpoH *cells, even at a low temperature (30°C) [10]. Therefore, we examined the chaperone activities at 25°C of AgsA and the N- or C-terminal truncated mutants for lysozyme (Table 1 and Additional file 1, Table S1). The addition of 10 μM AgsA, ΔN11 or ΔN17 to 10 μM DTT-denatured lysozyme reduced the turbidity due to aggregated lysozyme by approximately 10% compared to the turbidity observed for lysozyme alone at 25°C. ΔC11 showed weaker chaperone activity, since it was required at a higher concentration (20 μM) in order to prevent the aggregation of lysozyme at 25°C. We observed effective chaperone activity for 20 μM AgsA and the N-terminal truncated mutants at 37°C and 50°C. However, ΔC11 did not show any chaperone activity at these temperatures. Moreover, co-aggregation of ΔC11 with lysozyme (data not shown) resulted in more than a twofold increase in turbidity compared to lysozyme alone. These data suggested that the oligomeric structure or the C-terminal 11 AA was important for the chaperone activity of AgsA preventing the aggregation of lysozyme. Since AgsA could form stable oligomeric structures at 25°C, the important region(s) for its chaperone activity with lysozyme appeared to be localised to the outer surface of the oligomeric complex. This region might contribute to the *in vivo *chaperone activity of AgsA that was observed in the Δ*rpoH *mutant at 30°C.

**Table 1:** Percentage of turbidity^a ^of the DTT-denatured substrates

	lysozyme			insulin		
	
	25°C		50°C	25°C		50°C
	
	10 μM	20 μM	20 μM	10 μM	20 μM	20 μM
AgsA	12.3 ± 0.7	2.6 ± 0.2	2.9 ± 0.4	123.0 ± 0.4	119.7 ± 4.7	4.6 ± 0.3
ΔN11	8.8 ± 0.07	1.5 ± 0.2	1.1 ± 0.2	149.2 ± 7.4	132.8 ± 5.9	1.1 ± 0.3
ΔN17	10.0 ± 0.3	1.7 ± 0.2	5.0 ± 0.6	119.4 ± 0.6	11.1 ± 0.4	0.5 ± 0.3
ΔC11	114.2 ± 3.2	12.9 ± 0.4	231.1 ± 1.4	51.1 ± 1.3	1.5 ± 0.2	0.8 ± 0.08

^a^The percentage of turbidity showed the ratio of the turbidity of the DTT-denatured lysozyme (10 μM) or insulin (70 μM) with the indicated concentration of AgsA or its mutants to the turbidity of the DTT-denatured lysozyme or insulin alone (for details, see the Materials and Methods section). Values are the mean ± SD obtained from 3 independent experiments.

**Table 2:** Percentage of turbidity^a ^of the heat-denatured substrates

	MDH		CS		
	
	50°C	60°C	42°C	50°C	60°C
AgsA	3.2 ± 1.3	6.0 ± 0.3	0.4 ± 0.7	0.1 ± 0.1	2.7 ± 0.7
ΔN11	5.9 ± 1.5	40.1 ± 3.0	1.2 ± 2.1	0.6 ± 0.2	0.7 ± 0.3
ΔN17	87.3 ± 1.5	103.0 ± 2.4	0.6 ± 0.5	0.3 ± 0.2	1.9 ± 0.5
ΔC11	5.0 ± 2.6	81.5 ± 0.8	2.7 ± 2.5	39.8 ± 5.2	193.7 ± 15.3

^a^The percentage of turbidity showed the ratio of the turbidity of heat-denatured MDH (5 μM) or CS (1.5 μM) with 10 μM AgsA or its mutants to the turbidity of heat-denatured MDH or CS alone (for details, see the Materials and Methods section). Values are the mean ± SD obtained from 3 independent experiments.

**Figure 4:**
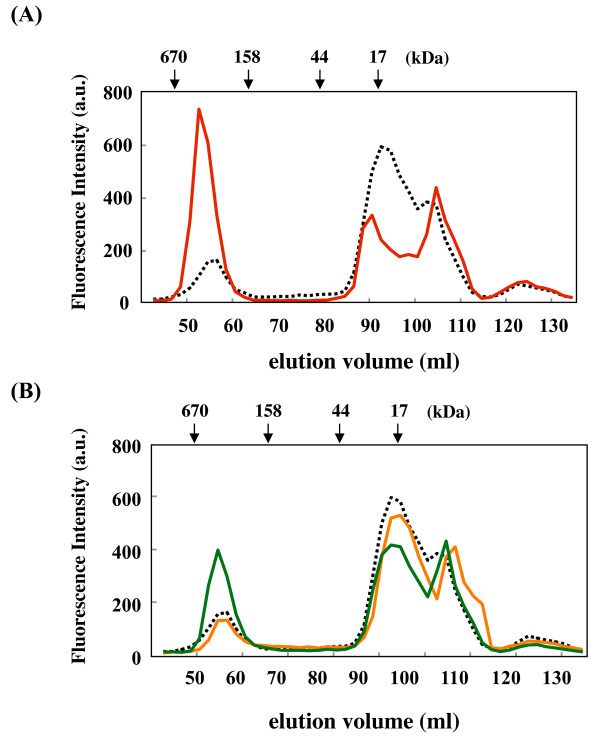
**Interaction denatured insulin with AgsA and/or ΔC11**. (A) Insulin (10 μM) was denatured in the presence of AgsA (50 μM) at 25°C or 50°C, and then analysed using size exclusion chromatography at 25°C. (B) Insulin (10 μM) was denatured in the presence of ΔC11 (50 μM) or AgsA (25 μM) and ΔC11 (25 μM) at 25°C, and then analysed using size exclusion chromatography. An aliquot of each fraction (200 μl) was excited at 490 nm and the fluorescence intensity was determined at 525 nm. The retention volume of the protein molecular weight standards is shown above the panel. Broken black line, insulin was denatured with AgsA at 25°C; red solid line, insulin was denatured with AgsA at 50°C; yellow solid line, insulin was denatured with ΔC11 at 25°C and green solid line, insulin was denatured with AgsA and ΔC11 at 25°C.

Interestingly, when DTT-denatured insulin (70 μM) was used as the substrate for AgsA or ΔC11 (Table 1 and Additional file 2, Table S2), ΔC11 significantly prevented the aggregation of insulin at every temperature examined. However, AgsA showed little or no chaperone activity at 25°C and 37°C. ΔN17 enhanced chaperone activity for insulin and significantly prevented its aggregation at 25°C and 37°C. AgsA and all truncated mutants demonstrated significant chaperone activity for insulin at 50°C. These results suggest that the important region(s) for chaperone activity with insulin are located inside the oligomeric complex. Destabilization of the oligomeric complex by C- or N-terminal truncations may expose this region(s) to the outside, even at low temperatures. We further examined the interaction between denatured insulin and AgsA or ΔC11 using SEC (Figure 4A). Since insulin has a small molecular weight (insulin A chain 2.3 kDa; insulin B chain 3.4 kDa), it was difficult to determine the eluted fraction using sodium dodecyl sulfate-polyacrylamide gel electrophoresis (SDS-PAGE). Therefore, we introduced a fluorescein label onto insulin in order to monitor its elution profile. In the following experiments 10 μM of denatured fluorescein-labelled insulin could not form large aggregates and was eluted in the small molecular fractions (data not shown). When insulin was denatured by DTT in the presence of AgsA at 25°C, almost all of the fluorescent insulin was eluted in the small molecular fractions. When the same experiment was carried out at 50°C, significant amounts of insulin were co-eluted with AgsA. These data are consistent with our hypothesis that the important region(s) for the chaperone activity of AgsA to insulin are located inside the oligomeric complex. However, we could not observe a significant interaction between denatured insulin and ΔC11 at 25°C (Figure 4B). The interaction between denatured insulin and ΔC11 could be weak and this complex may dissociate during SEC.

We also examined the chaperone activity of AgsA and its truncated mutants by using heat-denatured MDH and CS (Table 2 and Additional files 3 and 4, Tables S3 and S4). AgsA (10 μM) suppressed the aggregation of heat-denatured MDH (5 μM) at 50°C and 60°C (Table 2 and Additional file 3, Table S3). There was a significant reduction of chaperone activity in ΔN11 at 60°C. ΔN17 did not show any significant chaperone activity with MDH at 50°C or 60°C, indicating that the N-terminal 17 AA residues contain a crucial region for the prevention of MDH aggregation. ΔC11 prevented the aggregation of MDH at 50°C, although it displayed less activity than wild type AgsA. However, ΔC11 did not show any significant chaperone activity at 60°C. These results suggest that the chaperone activity of the dimer form of ΔC11 may be sufficient to prevent the aggregation of denatured MDH at 50°C. In contrast, the oligomeric complex may be necessary for highly denatured MDH at 60°C.

AgsA (10 μM) prevented the aggregation of CS (1.5 μM) from 42-60°C (Table 2 and Additional file 4, Table S4). In contrast to MDH, ΔN11 and ΔN17 possessed approximately the same level of chaperone activity as the wild type protein. Therefore, the N-terminal 17 AA residues of AgsA do not appear to have a crucial function for the prevention of CS aggregation. ΔC11 (10 μM) suppressed the aggregation of CS at 42°C. However, we observed a reduction in its chaperone activity in response to increases in the temperature. The ability of ΔC11 to suppress CS aggregation was completely lost at 60°C and co-aggregation was observed for both proteins (data not shown), as well as the case using lysozyme as the substrate. Thus, the chaperone activity of the dimeric form of ΔC11 was sufficient for locally denatured CS although the oligomeric complex seemed to be required for chaperone activity with highly denatured CS as well as MDH.

**Figure 5:**
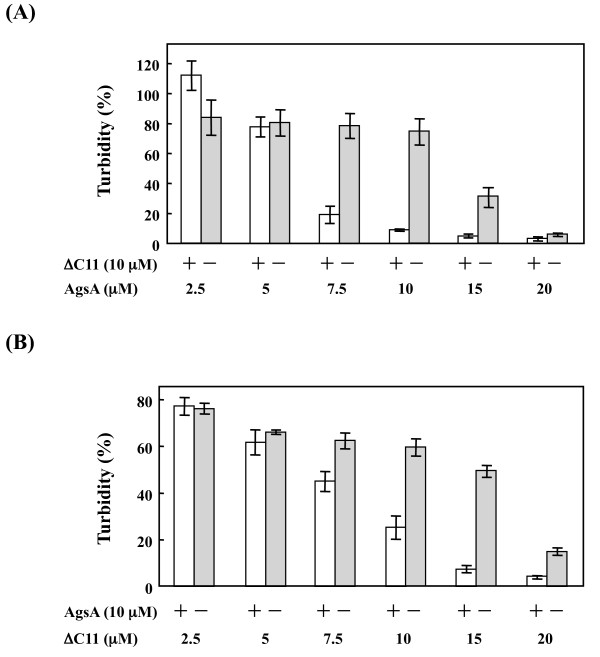
**Cooperative effect of AgsA and ΔC11 on aggregation prevention**. (A) Lysozyme (10 μM) was denatured by 20 mM DTT with the indicated concentration of AgsA in the presence or absence of 10 μM ΔC11 at 37°C for 30 min. (B) Insulin (70 μM) was denatured by 20 mM DTT with the indicated concentration of ΔC11 in the presence or absence of 10 μM AgsA at 25°C for 40 min. The percentage of turbidity (%) showed the ratio of the turbidity of the DTT-denatured lysozyme or insulin with AgsA and/or ΔC11 to the turbidity of the DTT-denatured lysozyme or insulin alone (for details, see the Materials and Methods section). Error bars show the SD from 3 different experiments.

Deletion of the C-terminal 11 AA caused the destabilization of the oligomeric structure (Figure 3) although the V/IXI/V motif, which is an important region for the formation of oligomeric sHsps, was preserved. Therefore, the addition of ΔC11 to AgsA might change the dynamic equilibrium between both dimer and oligomeric complex and overall chaperone activity. To investigate this possibility, equal amounts of AgsA and ΔC11 were mixed together (Additional file 5, Table S5). The mixture of AgsA and ΔC11 did not increase the turbidity in the absence of substrate by aggregation at our assayed temperatures (data not shown). Only half the amount of AgsA or ΔC11 required for efficient chaperone activity was used in the mixing experiment. For example, 60.2% lysozyme aggregated in the presence of 10 μM AgsA compared to lysozyme alone and 10 μM ΔC11 co-aggregated with denatured lysozyme, increasing the turbidity to 228.5% at 37°C. Interestingly, a mixture of 10 μM AgsA and ΔC11 reduced the aggregation of lysozyme to 8.9% at 37°C. This mixture also efficiently prevented the aggregation of insulin, and heat-denatured MDH and CS. Given these results, we then performed titration experiments by changing the ratios of AgsA to ΔC11 in the mixture using lysozyme as a substrate (Figure 5A). AgsA prevented the aggregation of lysozyme. However, ΔC11 was unable to prevent its aggregation above 37°C. So, the cooperative effect on chaperone activity with lysozyme was examined by changing the amount of AgsA in the presence or absence of 10 μM ΔC11 at 37°C. Although 20 μM AgsA was required to prevent the aggregation of lysozyme, 10 μM AgsA showed significant chaperone activity with lysozyme when ΔC11 was present. In contrast to lysozyme, ΔC11 was required to prevent the aggregation of insulin at low temperatures. The cooperative effect on chaperone activity to insulin was examined by changing the amount of ΔC11 in the presence or absence of 10 μM AgsA at 25°C (Figure 5B). We found that 15 μM ΔC11 effectively prevented the aggregation of insulin when AgsA was present, though 20 μM ΔC11 was required for significant chaperone activity to lysozyme. These data clearly demonstrate that AgsA and ΔC11 can interact with and activate each other, cooperatively.

We further analysed the cooperative effect of mixing AgsA and ΔC11 on their interaction with insulin using SEC (Figure 4B). In the case of denatured insulin in the presence of AgsA and ΔC11 at 25°C, a significant amount of insulin was eluted in the fractions that corresponded to the AgsA-insulin complex observed in Figure 4A. These data suggest that ΔC11 may affect the status of oligomeric complexes of AgsA and denatured insulin could incorporate into the AgsA oligomeric complexes.

### Determination of the interaction between AgsA and ΔC11

**Figure 6:**
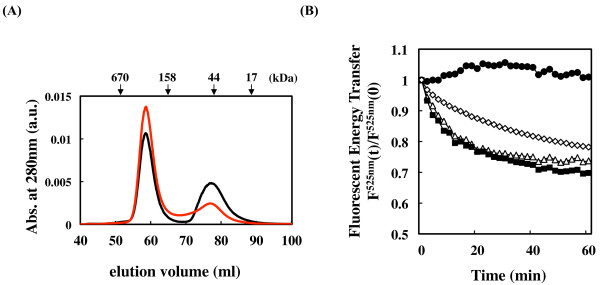
**Measurement of the interaction between AgsA and ΔC11**. (A) Equal amounts (50 μM) AgsA and ΔC11 were mixed and immediately (Black line) or after 1 h incubation at 25°C (red line) analysed using size exclusion chromatography at 25°C. The retention volume of the protein molecular weight standards is shown above the panel. (B) Fluorescein-labelled AgsA and rhodamine-labelled AgsA (solid circles), fluorescein-labelled ΔC11 and rhodamine-labelled AgsA (open diamond), fluorescein-labelled AgsA and rhodamine-labelled ΔC11 (open triangles), and fluorescein-labelled ΔC11 and rhodamine-labelled ΔC11 (solid squares) were mixed and incubated for the subunit exchange reaction at 25°C. The rate of subunit exchange is reflected by the time-dependent change for the emission fluorescence intensity of fluorescein recorded at 525 nm [F^525 nm^(t)/F^525 nm^(0)].

To monitor whether ΔC11 could change the dynamic equilibrium between the dimer and the oligomeric complexes of AgsA, we used SEC and fluorescence resonance energy transfer (FRET) analysis to observe the interaction between AgsA and ΔC11 at 25°C (Figure 6A and 6B). The cooperative effect of AgsA and ΔC11 on the complex size was determined using SEC (Figure 6A). Equal amounts of AgsA and ΔC11 were mixed and then immediately separated by SEC. The chromatograph of the mixture showed 2 peaks, corresponding to AgsA (419 kDa) and ΔC11 (66 kDa). When mixtures were incubated at 25°C for 1 h and then separated using SEC, the size of the 66 kDa peak corresponding to ΔC11 was decreased and the size of the 419 kDa peak corresponding to AgsA was increased. These data suggest that ΔC11 could associate with the AgsA oligomeric complex and form hetero-oligomeric complexes.

We further examined the interaction between AgsA and ΔC11 using FRET analysis (Figure 6B). For this purpose, purified AgsA and ΔC11 were labelled with fluorescein (as the fluorescence donor) or rhodamine (as the fluorescence acceptor). A decrease in fluorescein fluorescence at 525 nm was not observed when fluorescein-labelled AgsA and rhodamine-labelled AgsA were mixed, indicating that there was no exchange reaction between the 2 labelled AgsA subunits at 25°C. However, a significant exchange reaction was observed between the 2 labelled AgsA subunits at 45°C, with a 40% decrease in fluorescence compared to only fluorescein-labelled AgsA after 40 min (data not shown). In contrast to AgsA, mixing the 2 populations of labelled ΔC11 resulted in a decrease in fluorescence at 25°C, indicating an exchange reaction bringing the 2 labelled ΔC11 subunits close to each other. Mixing fluorescein-labelled AgsA and rhodamine-labelled ΔC11 or fluorescein-labelled ΔC11 and rhodamine-labelled AgsA also resulted in a decrease in fluorescence. However, IbpB, which is an sHSP and a major *E. coli *cytosolic chaperone [6,7], did not exhibit a subunit exchange reaction with AgsA (Additional file 6, Figure S1). These data clearly indicated that ΔC11 could promote AgsA subunit exchange at 25°C.

Overall our data suggest that ΔC11 can change the dynamic equilibrium between the dimer and the oligomeric complexes of AgsA, therefore, the mixture showed significant chaperone activity.

## Discussion

sHsps are conserved across prokaryotes and eukaryotes, and most of these sHsps form large oligomeric complexes. Oligomeric sHsps exhibit reduced chaperone activity at low temperatures and were believed to be the inactive form. At high temperatures, sHsps de-oligomerise and show full chaperone activity. Therefore, the chaperone activity of sHsps was regulated by changing their oligomeric structure in response to changes in the surrounding temperature [29]. Interestingly, AgsA showed *in vivo *chaperone activity at 30°C [10]. Nevertheless, it could form a stable oligomeric structure at low temperatures as shown in Figure 3. Therefore, we examined the *in vitro *chaperone activity of AgsA at low temperatures. In addition, N- or C-terminal truncated AgsA were constructed to investigate the relationship between oligomer formation and chaperone activity.

Our data showed that truncations up to the 23^rd ^AA of the N-terminal arm or to the 11^th ^AA from the C-terminus extension demonstrated significant chaperone activity *in vivo *(Figure 2). Although purified ΔN23 possessed *in vitro *chaperone activity in high ionic strength conditions (more than 400 mM), it aggregated in our assay conditions preventing us from examining its activity. The predicted α-helix region from the 17^th ^to the 23^rd ^AA of the N-terminal arm of AgsA appeared to contain an important region for the correct assembly of the oligomeric structure. ΔN11 and ΔN17 could form oligomeric complexes at 25°C. However, they could not retain the large oligomeric structure at 42°C (Figure 3). Thus, this region appears to strengthen the AgsA oligomer structure. Since ΔC11 could not assemble in a large oligomer, the 11 AA after the V/IXI/V motif also appear to have an important function in maintaining the AgsA oligomeric complex.

We further examined the *in vitro *chaperone activity of the N- or C-terminal truncated mutants. Our results showed that the N-terminal 17 AA of AgsA was a crucial region in preventing the aggregation of MDH, but not of other substrates (Table 2). It has been demonstrated that the N-terminal arm of sHsps is an important region for chaperone activity and substrate specificity using chimeras of all or part of the non-conserved N-terminal arm from pea Hsp18.1 and wheat Hsp16.9, and by cross-linking experiments between pea Hsp18.1 and its substrates [21,30]. The data presented in this study support these results; the N-terminal 17 AA of AgsA was an important region for its chaperone activity on MDH. ΔC11 only demonstrated chaperone activity with limited substrates and conditions, *e.g*. denatured insulin and partially denatured MDH and CS (Tables 1 and 2). However it possessed little or no chaperone activity towards denatured lysozyme or strongly denatured MDH and CS. These results suggest that the ability of AgsA to form oligomeric complexes may be important for its chaperone activity with strongly denatured protein substrates.

Interestingly, mixing of AgsA and ΔC11 compensated for the defects in their individual chaperone activities, *e.g*. AgsA did not show efficient chaperone activity towards denatured insulin at low temperatures, and ΔC11 showed weak chaperone activity towards denatured lysozyme and strongly denatured MDH and CS (Additional file 5, Table S5). Titration experiments also showed that the chaperone activity increased in a positive-synergistic manner of AgsA or ΔC11 (Figure 5). These data strongly suggest that AgsA and ΔC11 can interact with each other and activate their chaperone activity cooperatively. Inter-subunit exchanges among AgsA complexes were not detected using FRET analysis at 25°C, indicating that AgsA can form a stable oligomeric structure (Figure 6B). However, inter-subunit exchanges between AgsA and ΔC11 complexes were observed at this temperature. These results suggest that the dynamic equilibrium between the dimer and the oligomeric complex is important for the chaperone activity of AgsA with wide substrates spectrum. Does such an association exist in the cell? Interestingly, sHsps are preserved in many eukaryotes, *i.e*. 16 in *Caenorhabditis elegans *and 10 in *Homo sapiens *[14]. Therefore, we cannot exclude the possibility that the interaction among different sHsps could modulate their chaperone activity. Mixing of different sHsps may suggest us a possibility of the cellular functions of eukaryotic sHsps. It has been reported that human sHsps may contribute directly to muscle diseases, certain cancers and neurodegenerative diseases, such as Alzheimer's and Parkinson's disease [31]. Increasing our knowledge of sHSPs by investigating the quality control mechanisms for the cellular targets of AgsA may help us to understand the causes of such diseases.

## Conclusions

AgsA requires multiple regions for its chaperone activity: important region(s) for denatured lysozyme are located outer surface of the oligomeric complex, for denatured insulin are located inside of the oligomeric complex and for denatured MDH are located in a 17 AA region of the N-terminal arm. ΔC11 could not form large oligomeric complexes and did not show any chaperone activity towards strongly denatured substrates. Therefore, the ability to form large oligomeric complex appears to be important for the chaperone activity of AgsA. The mixing of AgsA and ΔC11 caused inter-subunit exchange in individual complex, compensated for the defects in individual chaperone activities demonstrating good chaperone activity for all of the substrates and temperatures examined. These results indicate that the dynamic equilibrium between the dimer and the oligomeric complex may be important for the efficient chaperone activity of AgsA.

## Methods

### Bacterial strains, plasmids, and growth conditions

*Escherichia coli* K-12 strain DH5αZ1 [F^-^, Φ80d*lacZ*ΔM15, Δ(*lacZYA-argF*)U169, *deoR, recA1, endA1, hsdR17*(r_K_^-^, m_K_^+^), *phoA, supE44,* λ^-^, *thi-1, gyrA96, relA1 tetR lacI^q ^*Spec^r^] [32], BB7224 [F^- ^*araD* Δ(*argF-lac*)U169 *rpsL relA flbB deoC ptsF rbsR* Δ*rpoH*::Km *suhX401*] [27], CS5262 (BB7224 carrying the Lac repressor producing plasmid pBB528) [10], and its derivatives were used in our experiments. Cells were grown in Luria-Bertani (LB) medium at the indicated temperatures under aerobic conditions. Ampicillin (50 μg/ml), chloramphenicol (20 μg/ml), and kanamycin (20 μg/ml) were added to the medium.

### Databases, multiple sequence alignment, and modeling of protein structure

Protein sequences were obtained from GenBank or the Protein Data Bank (PDB) by an Entrez cross-database search at the National Center for Biotechnology Information (National Institutes of Health, USA). Multiple sequence alignments were constructed with the Parallel PRRN program (Kyoto University Bioinformatics Center, Japan) [33]. The three-dimensional structure of AgsA was predicted using the Geno3 D server, which is an automated protein modeling Web server to generate three-dimensional protein models (PBIL, Lyon, France).

### Construction of His-tagged recombinant AgsA or IbpB overexpression plasmids

The *agsA *gene coding region was amplified from *Salmonella enterica *serovar Typhimurium χ3306 chromosomal DNA using the primers his-tag AgsA F and his-tag AgsA R (Table 3) to construct the His-tagged AgsA overproducing plasmid (pUHE212-1 *agsA*) [10]. The *ibpB *gene coding region was amplified from pBB572 using the primers his-tag IbpB F and his-tag IbpB R (Table 3) to construct the His-tagged IbpB overproducing plasmid (pUHE212-1 *ibpB*) [34]. The amplified fragment was digested with *Bam*HI and *Hin*dIII and then cloned into pUHE212-1 [35].

**Table 3:** Primers used in this study

PCR primer	Sequence
his-tag AgsA F	GTTAGGATCCGCACTCAGAACCTTGTCAGC
his-tag AgsA R	ATTAAGCTTATGATTTGTGTTCAATCGCC
ΔN7 F	GAGGATCCTCAGCACTTCCCGTGTTTGCTG
ΔN11 F	CAGCATCCGTGTTTGCTGATTCTCTTTTC
ΔN17 F	GATGGATCCTTCTCTGACCGTTTCAACCG
ΔN23 F	CGTGGATCCCGTATTGATAGACTTTTCAGTC
ΔN27 F	TTGGATCCCTTTTCAGTCAATTAACAGGAG
ΔC11 R	GTTAAGCTTATATGGCAATTTTTTTCGGTTTCTC
ΔC24 R	GGAAGCTTCTGGTAAATCTCGACCAAG
his-tag IbpB F	GAAGGATCCATGACTATGCGTAACTTCG
his-tag IbpB R	TAGAAGCTTAGCTATTTAACGCGGGACG

### N- or C-terminal truncated AgsA overexpression plasmids

N- or C-terminal truncated *agsA *genes were amplified from pUHE212-1 *agsA *using the primers shown in Table 3. ΔN7 F and his-tag AgsA R were used for the N-terminal 7 AA truncation of the *agsA *gene, ΔN11 F and his-tag AgsA R for the N-terminal 11 AA truncation, ΔN17 F and his-tag AgsA R for the N-terminal 17 AA truncation, ΔN23 F and his-tag AgsA R for the N-terminal 23 AA truncation, ΔN27 F and his-tag AgsA R for the N-terminal 27 AA truncation, his-tag AgsA F and ΔC11 R for the C-terminal 11 AA truncation, and his-tag AgsA F and ΔC24 R for the C-terminal 24 AA truncation. The amplified fragment was digested with *Bam*HI and *Hin*dIII and then cloned into pUHE212-1.

### *In vivo *chaperone activity of N- or C-terminal truncated AgsA

Chaperone activity was determined by measuring the amount of aggregated protein in the Δ*rpoH *mutant (CS5262). The AgsA overproducing plasmid (pUHE212-1 *agsA*) was transformed in CS5262. N- or C-terminal truncated AgsA overproducing plasmids (pΔN7, N-terminal 7 AA truncated AgsA; pΔN11, N-terminal 11 AA truncated AgsA; pΔN17, N-terminal 17 AA truncated AgsA; pΔN23, N-terminal 23 AA truncated AgsA; pΔN27, N-terminal 27 AA truncated AgsA; pΔC11, C-terminal 11 AA truncated AgsA; and pΔC23, C-terminal 23 AA truncated AgsA) were also transformed in CS5262. Isolation of total proteins and aggregated proteins was performed as previously described with minor modifications [10,27]. Cells were grown in 10 ml LB medium with 1 mM IPTG for 4 h at 30°C and then shifted to 42°C for 1 h. After heat treatment, bacterial cultures were rapidly cooled on ice and centrifuged for 10 min at 5000 × *g *at 4°C to harvest the cells. Pellets were resuspended in 80 μl buffer A (10 mM potassium phosphate buffer pH 6.5, 1 mM EDTA, 20% (w/v) sucrose, 1 mg/ml lysozyme) and incubated for 30 min on ice. Spheroplasts were destroyed by the addition 720 μl buffer B (10 mM potassium phosphate buffer pH 6.5, 1 mM EDTA) and sonicated with an Astrason XL2020 ultrasonic processor (microtip, level 3, 50% duty, 10 s) while cooling. The insoluble fraction from total proteins was isolated by centrifugation at 17,000 × *g *for 5 min at 4°C. The pellet fractions were frozen, resuspended in 800 μl buffer B by sonication, and centrifuged (17,000 × *g*, 5 min, 4°C). The washed pellet fractions were again resuspended in 640 μl buffer B by brief sonication; afterwards, 160 μl of 10% (v/v) NP40 was added, and the aggregated proteins were isolated by centrifugation (17,000 × *g*, 5 min, 4°C). This washing procedure was repeated to complete removal of contaminating membrane proteins. NP40-insoluble pellets were washed with 800 μl buffer B and resuspended in 200 μl buffer B by brief sonication. Quantification of the amount of total proteins and aggregated proteins was performed using the Bradford assay reagent (Bio-Rad, Hercules, CA, USA) with bovine serum albumin (BSA) as a standard.

### SDS-PAGE and protein quantification

Each 10 μg of total proteins from AgsA- or truncated AgsA-overproducing strains was analyzed by using 15% SDS-polyacrylamide gels according to the method of Laemmli and then stained with Coomassie brilliant blue R250 [36]. The stained gels were digitized using a LAS-4000miniEPUV lumino-image analyzer (Fuji Film, Tokyo, Japan), and the level of AgsA and its truncated mutants were quantified by using Multi Gauge Ver3.0 software (Fuji Film, Tokyo, Japan).

### Purification of His-tagged recombinant proteins

Overexpression of the recombinant proteins was induced by adding 1 mM IPTG to mid-log phase *E. coli *cells, and the culture was continued at 37°C for 3 h. The cells were then harvested by centrifugation (5,000 × *g*, 30 min, 4°C) and resuspended in 20 mM Tris-HCl (pH 8.0) containing 300 mM NaCl, 20 mM imidazole and 6 M urea. The suspension was sonicated using an Astrason XL2020 ultrasonic processor and then incubated at 30°C for 1 h to denature the proteins. The resultant cell extract was centrifuged at 10,000 × *g *for 30 min to remove unbroken cells. The supernatant was loaded onto a Ni-NTA agarose column (Qiagen, CA, USA). To renature the denatured proteins in the column, the urea concentration in the buffer was gradually reduced from 6 to 0 M, as described by Colangeli *et al*. [37]. The column was then washed with 20 mM Tris-HCl (pH 8.0) containing 300 mM NaCl and 20 mM imidazole. Proteins bound to the column were eluted with a linear gradient of 0-1.0 M imidazole in 20 mM Tris-HCl (pH 8.0) containing 300 mM NaCl. Peak fractions were diluted ten-fold with 20 mM Tris-HCl buffer (pH 8.0) containing 1 mM EDTA and loaded onto an Econo-Pac high-Q cartridge (Bio-Rad Co., CA, USA). The N-his recombinant protein was eluted with a linear gradient of 0-1.0 M NaCl in 20 mM Tris-HCl buffer (pH 8.0) containing 1 mM EDTA, and the peak fractions were frozen at -80°C until use.

### Gel filtration

Gel filtration chromatography was performed using a Superdex™ 200 XK 16/70 gel filtration column (GE Healthcare, Buckinghamshire, UK) pre-equilibrated with 20 mM Tris-HCl buffer (pH 8.0) containing 1 mM EDTA and 100 mM NaCl. The column pre-equilibrated with 40 mM HEPES buffer (pH 7.5) was used to measure the interaction between denatured insulin and AgsA and/or ΔC11. Separation was performed on a BioLogic DuoFlow Chromatography system (Bio-Rad Co., CA, USA) or AKTAprime plus (GE Healthcare, Buckinghamshire, UK) at the indicated temperatures and a flow rate of 1.0 ml/min. The following mass standards (Bio-Rad Co., CA, USA) were used to calibrate the column: thyroglobulin (669 kDa), bovine gamma globulin (158 kDa), chicken ovalbumin (44 kDa), equine myoglobin (17 kDa) and vitamin B12 (1.4 kDa). Plotting the logarithms of the known molecular weights of mass standards vs their respective Ve/Vo values produced a linear calibration curve that was used to calculate the molecular weights of AgsA and the truncated proteins.

### Assay of *in vitro *chaperone activity

The chaperone activity of wild type and truncated AgsA towards chemically denatured substrates was measured using lysozyme from chicken egg white (Wako Pure Chemical Co., Osaka, Japan) and insulin from bovine pancreas (Nacalai Tesque, Kyoto, Japan). Lysozyme (10 μM) in 20 mM Tris-HCl buffer (pH 8.0) containing 1 mM EDTA and 100 mM NaCl was incubated with 20 mM DTT and different amounts of AgsA or truncated AgsA. Reaction mixtures without DTT were pre-incubated in a water bath at the indicated temperature for 10 min. After the addition of DTT, the samples were incubated for 90 min (25°C), 60 min (37°C and 42°C) or 30 min (50°C), and light scattering at 360 nm was monitored in a BioSpec-mini spectrophotometer (Shimadzu, Kyoto, Japan). Insulin (70 μM) in 40 mM HEPES buffer (pH 7.5) was incubated with 20 mM DTT and different amounts of wild type or truncated AgsA. Reaction mixtures without DTT were pre-incubated in a water bath at the indicated temperature for 10 min. After the addition of DTT, the samples were incubated for 30 min at the indicated temperature, and light scattering at 360 nm was monitored. Negative control reactions were performed as follows: (1) without substrate, with or without AgsA or truncated AgsA, in the presence or absence of DTT, or (2) with substrate and AgsA or truncated AgsA in the absence of DTT.

The chaperone activity of AgsA and truncated AgsA towards heat-denatured substrates was measured using L-malate dehydrogenase (Oriental Yeast Ind. Co., Tokyo, Japan) and citrate synthase from porcine heart (Sigma Chemical Co., MI, USA). MDH (5 μM) was incubated with different amounts of AgsA and truncated AgsA in 20 mM Tris-HCl buffer (pH 8.0) containing 1 mM EDTA and 100 mM NaCl. Reaction mixtures without MDH were kept in a water bath at the indicated temperature for 10 min. After the addition of MDH, the samples were incubated at the indicated temperature for 30 min, and light scattering at 360 nm was monitored. CS (1.5 μM) was incubated with different amounts of AgsA and truncated AgsA in 40 mM HEPES buffer (pH 7.5). Reaction mixtures without CS were kept in a water bath at the indicated temperature for 10 min. After the addition of AgsA and truncated AgsA, the samples were incubated for 40 min (42°C and 50°C) or 30 min (60°C), and light scattering at 360 nm was monitored. The percentage of turbidity was calculated using the following formula: *A*_360 _(substrate + wild type and/or truncated AgsA) */A*_360 _(substrate alone) × 100.

### Construction of fluorescently labelled proteins

Insulin (100 μM) was labelled with 200 μM NHS-fluorescein (Thermo Fisher Scientific Inc., IL, USA) in 20 mM phosphate buffer (pH 8.0) containing 150 mM NaCl for 1 h at room temperature. Unreacted fluorescein was separated from the labelled insulin using a 5 ml HiTrap Desalting Column (GE Healthcare, Buckinghamshire, UK) with 40 mM HEPES buffer (pH 7.5). AgsA, ΔC11 and IbpB (100 μM) were labelled with 400 μM NHS-fluorescein in 20 mM phosphate buffer (pH 8.0) containing 150 mM NaCl for 1 h at room temperature. AgsA, ΔC11 and IbpB (100 μM) were also labelled with 400 μM NHS-rhodamine (Thermo Fisher Scientific Inc., IL, USA) in 20 mM phosphate buffer (pH 7.2) containing 150 mM NaCl for 1 h at room temperature. Unreacted fluorescein or rhodamine was separated from the labelled proteins by dialysis with 20 mM Tris-HCl buffer (pH 8.0) containing 1 mM EDTA and 100 mM NaCl. The final concentration of the protein preparations was measured and then separated on SDS-PAGE. The absence of free fluorescent label was confirmed by fluorescence imaging of the unstained gels.

### Subunit-exchange studies by FRET

Interactions between AgsA and C-terminal truncated AgsA or between AgsA and IbpB were determined using a FRET method based on the subunit-exchange reaction. To initiate the subunit-exchange reaction, 10 μM fluorescein- and 10 μM rhodamine-labelled protein in 20 mM Tris-HCl buffer (pH 8.0) containing 1 mM EDTA and 100 mM NaCl were mixed together in a microtiter plate at 25°C. The samples were subsequently excited at 490 nm and the emission intensity was determined at 525 nm. The changes in the donor fluorescence intensity were calculated as a function of time, *F *(*t*), from that at zero time, *F *(0). All fluorescence spectra were recorded using a Gemini XPS Microplate Spectrofluorometer (Molecular Devices, CA, USA).

## Abbreviations

AA: amino acids; bp: base pairs; Abs.: absorbance; a.u.: arbitrary unit; CS: citrate synthase; kDa: kilodalton; DTT: dithiothreitol; FRET: fluorescence resonance energy transfer; MDH: malate dehydrogenase; SEC: size exclusion chromatography; SD: standard division; sHSP: small heat shock protein: IPTG: isopropyl-beta-D-thiogalactopyranoside.

## Authors' contributions

TT contributed with planning the experimental design, performing the experiments, and writing the manuscript. AT and HN contributed with supervising and writing the manuscript. All authors read and approved the final manuscripts.

## Supplementary Material

Additional file 1 **Table S1**. Percentage of turbidity of DTT-denatured lysozyme. Click here for file

Additional file 2 **Table S2**. Percentage of turbidity of DTT-denatured insulin. Click here for file

Additional file 3 **Table S3**. Percentage of turbidity of heat-denatured MDH. Click here for file

Additional file 4 **Table S4**. Percentage of turbidity of heat-denaturfed CS. Click here for file

Additional file 5 **Table S5**. Cooperative effect of AgsA and ΔC11 on the aggregation prevention of several substrates. Click here for file

Additional file 6 **Figure S1**. Measurement of subunit exchange between AgsA and IbpB. Click here for file
