# Supplementary material for: Investigation of the chaperone function of the small heat shock protein — AgsA
Source: BMC Biochem. 2010 Jul 24;11:27. doi: 10.1186/1471-2091-11-27 (PMC2920228; doi:10.1186/1471-2091-11-27)
Supplement: Additional file 1 — Table S1. Percentage of turbidity of DTT-denatured lysozyme. [file 1471-2091-11-27-S1.DOC]

## Table S1 - Percentage of turbidity^a^ of DTT-denatured lysozyme

|  | 25˚C | | 37˚C | | 50˚C | |
| --- | --- | --- | --- | --- | --- | --- |
|  | 10 μM | 20 μM | 10 μM | 20 μM | 10 μM | 20 μM |
| AgsA | 12.3 ± 0.7 | 2.6 ± 0.2 | 60.2 ± 26.5 | 2.1 ± 0.3 | 38.6 ± 10.2 | 2.9 ± 0.4 |
| ΔN11 | 8.8 ± 0.07 | 1.5 ± 0.2 | 67.5 ± 3.3 | 1.6 ± 0.4 | 36.4 ± 2.0 | 1.1 ± 0.2 |
| ΔN17 | 10.0 ± 0.3 | 1.7 ± 0.2 | 68.9 ± 6.3 | 2.0 ± 0.5 | 106.3 ± 1.3 | 5.0 ± 0.6 |
| ΔC11 | 114.2 ± 3.2 | 12.9 ± 0.4 | 228.5 ± 2.9 | 254.0 ± 7.3 | 227.5 ± 1.0 | 231.1 ± 1.4 |

^a^The percentage of turbidity shows the ratio of the turbidity of DTT-denatured lysozyme (10 μM) with the indicated concentration of AgsA or its mutants to the turbidity of DTT-denatured lysozyme alone (for details, see the Materials and Methods section). Values are the mean ± SD obtained from 3 independent experiments.
