# Supplementary material for: Investigation of the chaperone function of the small heat shock protein — AgsA
Source: BMC Biochem. 2010 Jul 24;11:27. doi: 10.1186/1471-2091-11-27 (PMC2920228; doi:10.1186/1471-2091-11-27)
Supplement: Additional file 2 — Table S2. Percentage of turbidity of DTT-denatured insulin. [file 1471-2091-11-27-S2.DOC]

## Table S2 - Percentage of turbidity^a^ of DTT-denatured insulin

|  | 25˚C | | 37˚C | | 50˚C | |
| --- | --- | --- | --- | --- | --- | --- |
|  | 10 μM | 20 μM | 10 μM | 20 μM | 5 μM | 10 μM |
| AgsA | 123.0 ± 0.4 | 119.7 ± 4.7 | 88.3 ± 3.3 | 60.0 ± 10.2 | 37.9 ± 1.4 | 4.6 ± 0.3 |
| ΔN11 | 149.2 ± 7.4 | 132.8 ± 5.9 | 65.6 ± 1.9 | 19.5 ± 1.7 | 30.3 ± 1.0 | 1.1 ± 0.3 |
| ΔN17 | 119.4 ± 0.6 | 11.1 ± 0.4 | 19.7 ± 2.4 | 1.4 ± 0.1 | 14.1 ± 3.9 | 0.5 ± 0.3 |
| ΔC11 | 51.1 ± 1.3 | 1.5 ± 0.2 | 5.1 ± 0.7 | 0.4 ± 0.3 | 5.5 ± 0.9 | 0.8 ± 0.08 |

^a^The percentage of turbidity shows the ratio of the turbidity of DTT-denatured insulin (70 μM) with the indicated concentration of AgsA or its mutants to the turbidity of DTT-denatured insulin alone (for details, see the Materials and Methods section). Values are the mean ± SD obtained from 3 independent experiments.
