# Supplementary material for: Investigation of the chaperone function of the small heat shock protein — AgsA
Source: BMC Biochem. 2010 Jul 24;11:27. doi: 10.1186/1471-2091-11-27 (PMC2920228; doi:10.1186/1471-2091-11-27)
Supplement: Additional file 3 — Table S3. Percentage of turbidity of heat-denatured MDH. [file 1471-2091-11-27-S3.DOC]

## Table S3 - Percentage of turbidity^a^ of heat-denatured MDH

|  | 50˚C | | | 60˚C | | |
| --- | --- | --- | --- | --- | --- | --- |
|  | 2.5 μM | 5 μM | 10 μM | 2.5 μM | 5 μM | 10 μM |
| AgsA | 15.6 ± 0.7 | 5.6 ± 1.5 | 3.2 ± 1.3 | 42.5 ± 5.8 | 13.3 ± 0.4 | 6.0 ± 0.3 |
| ΔN11 | 78.2 ± 1.7 | 20.8 ± 0.7 | 5.9 ± 1.5 | 99.1 ± 2.4 | 77.5 ± 1.1 | 40.1 ± 3.0 |
| ΔN17 | 97.3 ± 1.0 | 88.9 ± 5.2 | 87.3 ± 1.5 | 108.7 ± 3.9 | 103.0 ± 0.8 | 103.0 ± 2.4 |
| ΔC11 | 66.9 ± 0.4 | 16.0 ± 0.5 | 5.0 ± 2.6 | 84.8 ± 3.9 | 78.8 ± 1.8 | 81.5 ± 0.8 |

^a^The percentage of turbidity shows the ratio of the turbidity of heat-denatured MDH (5 μM) with the indicated concentration of AgsA or its mutants to the turbidity of heat-denatured MDH alone (for details, see the Materials and Methods section). Values are the mean ± SD obtained from 3 independent experiments.
