# Supplementary material for: Investigation of the chaperone function of the small heat shock protein — AgsA
Source: BMC Biochem. 2010 Jul 24;11:27. doi: 10.1186/1471-2091-11-27 (PMC2920228; doi:10.1186/1471-2091-11-27)
Supplement: Additional file 4 — Table S4. Percentage of turbidity of heat-denaturfed CS. [file 1471-2091-11-27-S4.DOC]

## Table S4 - Percentage of turbidity^a^ of heat-denatured CS

|  | 42˚C | 50˚C | 60˚C | | |
| --- | --- | --- | --- | --- | --- |
|  | 10 μM | 10 μM | 2.5 μM | 5 μM | 10 μM |
| AgsA | 0.4 ± 0.7 | 0.1 ± 0.1 | 10.1 ± 1.3 | 5.7 ± 0.4 | 2.7 ± 0.7 |
| ΔN11 | 1.2 ± 2.1 | 0.6 ± 0.2 | n.d. | n.d. | 0.7 ± 0.3 |
| ΔN17 | 0.6 ± 0.5 | 0.3 ± 0.2 | 23.9 ± 8.1 | 5.1 ± 1.4 | 1.9 ± 0.5 |
| ΔC11 | 2.7 ± 2.5 | 39.8± 5.2 | 184.4 ± 32.7 | 180.9 ± 28.4 | 193.7 ± 15.3 |

^a^The percentage of turbidity shows the ratio of the turbidity of heat-denatured CS (1.5 μM) with the indicated concentration of AgsA or its mutants to the turbidity of heat-denatured CS alone (for details, see the Materials and Methods section). Values are the mean ± SD obtained from 3 independent experiments. n.d., not determined.
