# Supplementary material for: Investigation of the chaperone function of the small heat shock protein — AgsA
Source: BMC Biochem. 2010 Jul 24;11:27. doi: 10.1186/1471-2091-11-27 (PMC2920228; doi:10.1186/1471-2091-11-27)
Supplement: Additional file 5 — Table S5. Cooperative effect of AgsA and ΔC11 on the aggregation prevention of several substrates. [file 1471-2091-11-27-S5.DOC]

## Table S5 - Cooperative effect of AgsA and ΔC11 on the aggregation prevention of several substrates

|  | 37˚C | | 60˚C | |
| --- | --- | --- | --- | --- |
|  | Lysozyme | Insulin | MDH | CS |
|  | 10 μM | 10 μM | 2.5 μM | 2.5 μM |
| AgsA | 60.2 ± 26.5 | 109.6 ± 2.1 | 42.5 ± 5.8 | 10.1 ± 1.3 |
| ΔC11 | 228.5 ± 2.9 | 54.0 ± 1.1 | 84.8 ± 3.9 | 184.4 ± 32.7 |
| AgsA + ΔC11 | 8.9 ± 0.2 | 38.5 ± 0.3 | 18.5 ± 0.8 | 1.8 ± 1.0 |

Values were the percentage of turbidity shows the ratio of the turbidity of heat-denatured substrates (lysozyme, 10 μM; insulin, 70 μM; MDH, 5 μM; and CS, 1.5 μM) with the presence of the indicated concentration of AgsA and/or ΔC11 (for details, see the Materials and Methods section). Values are the mean ± SD obtained from 3 independent experiments.
