# Supplementary material for: Investigation of the chaperone function of the small heat shock protein — AgsA
Source: BMC Biochem. 2010 Jul 24;11:27. doi: 10.1186/1471-2091-11-27 (PMC2920228; doi:10.1186/1471-2091-11-27)
Supplement: Additional file 6 — Figure S1. Measurement of subunit exchange between AgsA and IbpB. [file 1471-2091-11-27-S6.DOC]

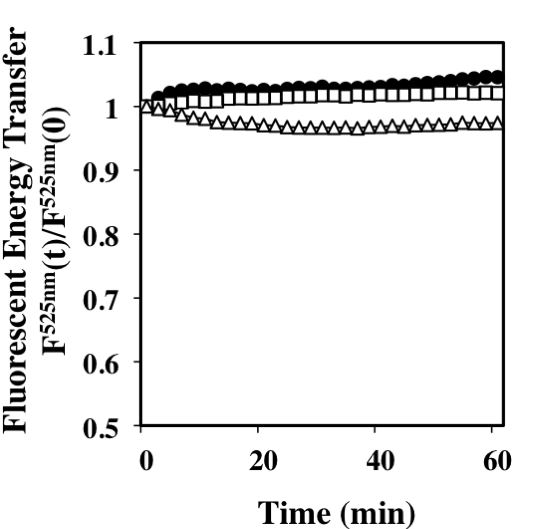


## Figure S1 - Measurement of subunit exchange between AgsA and IbpB

Fluorescein-labelled IbpB and rhodamine-labelled IbpB (solid circles), fluorescein-labelled AgsA and rhodamine-labelled IbpB (open squares), and fluorescein-labelled IbpB and rhodamine-labelled AgsA (open triangles) were mixed and incubated at 25°C. The rate of subunit exchange is reflected by the time-dependent change for the emission fluorescence intensity of fluorescein recorded at 525 nm [F^525nm^(t)/ F^525nm^(0)].
